# Effects of wheat bran replacement with pomegranate seed pulp on rumen fermentation, gas production, methanogen and protozoa populations of camel and goat rumen using competitive PCR technique: An in vitro study

**DOI:** 10.1002/vms3.1297

**Published:** 2023-10-21

**Authors:** Sanaz Jaberi Darmiyan, Mohammad Bagher Montazer Torbati, Mohammad Ramin, Seyed Ehsan Ghiasi

**Affiliations:** ^1^ Department of Animal Science, Faculty of Agriculture University of Birjand Birjand Iran; ^2^ Research Group of Environmental Stress in Animal Science, Faculty of Agriculture University of Birjand Birjand Iran; ^3^ Department of Animal Nutrition and Management Swedish University of Agricultural Sciences Umeå Sweden

**Keywords:** camel, competitive PCR, goat, methanogen, pomegranate seed pulp, protozoa

## Abstract

**Background:**

Microbial populations in the rumen play an essential role in the degradation of Cellulosic dietary components and in providing nutrients to the host animal.

**Objective:**

This study aims to detect the effect of pomegranate seed pulp (PSP) on rumen fermentation, digestibility and methanogens and the protozoa population (by competitive polymerase chain reaction [PCR]) of the camel and goat rumen fluid.

**Materials and methods:**

PSP was added to the experimental treatments and replaced by wheat bran (0%, 5% and 10%). Rumen fluid was collected from three goats and two camels according to the similarity of sex, breed, origin and time and used for three gas production studies. DNA extraction was performed by the RBB + c method, the ImageJ programme calculated band intensities (target and competing DNA), and line gradients were plotted based on the number of copies and intensity.

**Results:**

Our result showed that diets did not significantly affect the methanogen and protozoa population. Animal species affected microbial populations so that both populations in camels were less than goats. The production of gas and volatile fatty acids was not affected by diets. These two parameters and NH_3_ concentration and methane production in goats were higher than in camel. The pH of digested dry matter and microbial protein in camels was higher than in goats.

**Conclusions:**

Therefore, the competitive PCR technique is an effective method for enumerating rumen microbiota. This supplementation can be considered a strategy to achieve performance and environmental benefits.

## Graphical abstract

- The competitive PCR technique is an effective method for the enumeration of rumen microbiota.
- This supplementation can be considered a strategy to achieve performance and environmental benefits.

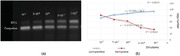

## 1 INTRODUCTION

The rumen has one of the most diverse microbial populations in nature, such as bacteria, archaea, protozoa, viruses and fungi. These populations play an essential role in providing agricultural products available to humans to consume as a nutrient‐dense food source by the anaerobic fermentation and degradation of dietary components, especially their ability to use and increase the productivity of cellulosic feeds providing nutrients to the host animal (Matthews et al., 2019).

During the rumen fermentation process, microorganisms convert plant carbohydrates into volatile fatty acids (VFAs such as propionate, acetate and butyrate), lactic acid, carbon dioxide, methane and hydrogen (Newbold & Ramos‐Morales, 2020). Methanogenic archaea are the primary consumers of hydrogen, which produces methane by combining CO_2_ and H_2_, and these are also involved in the production of VFA, especially acetate. Methane production by archaea represents an energy loss of about 2%–12% of gross energy intake, meaning not only is this energy no longer available for animal growth, lactation, maintenance or pregnancy, but it also contributes to global warming (Patra et al., 2017).

CH_4_ emissions from ruminant livestock contribute about 40%–45% of global greenhouse gas (GHG) emissions (McAllister et al., 2015). In order to increase access to hydrogen, methanogens may engage in a symbiotic relationship with rumen protozoa, which produce hydrogen via their hydrogenosomes. It has been estimated that approximately 37% of CH_4_ from ruminants is produced by protozoa‐associated methanogens (Bhatta et al., 2013). Elimination of the ciliate protozoa increases microbial protein supply by up to 30% and reduces CH_4_ production by up to 11% (Bhatta et al., 2013). Rumen microbial population composition is influenced by various factors, such as diet components, the number of feedings (Denman & McSweeney, 2006), dietary additives (Khejornsart et al., 2011), geographical location (Sundset et al., 2009) and animal impact (Wanapat, 2000).

Pomegranate (*Punica granatum* L.) is one of the most important fruit crops cultivated in the Middle East, Mediterranean, Caucasus and Indian subcontinents and central Asia. Iran, with an annual production of 1 million tons with 65,000 ha of pomegranate cultivation, is the leading exporter of pomegranate to the world (Hassani Moghaddam & Sepahvand, 2020). Pomegranate seed pulp (PSP) is a by‐product of the pomegranate juice industry, which includes its peel and seeds. It contains antioxidants, fats, anti‐inflammatory compounds, vitamin E, minerals, crude protein (CP), fibre and polyphenols and can be substituted for some feed components in animal nutrition (Aruna et al., 2016; Emami et al., 2015; Prakash & Prakash, 2011). Many studies suggest that the addition of pomegranate pulp to broiler chickens’ diet has a good effect on the growth rate, blood serum metabolites, immunological parameters and the quality of meat (Abdel Baset et al., 2020; Leontopoulos et al., 2021). Dietary inclusion of pomegranate peel extract improved animal growth, N retention, milk fatty acid profile and antioxidant capacities of the blood and rumen fluid and also decreased the ruminal total protozoa enumeration and population (Rajabi et al., 2017; Modaresi et al., 2011).

Competitive polymerase chain reaction (cPCR) assay is a quantitative molecular technique that enumerates susceptible targeted molecules. This approach is based on the competitive amplification of a specific target sequence together with an internal standard, the concentration of which is known (Zimmermann & Mannhalter, 1996). It has been used in the detection of pathogenic fungi, microorganisms and viruses (Sidhu et al., 1999) or for analysing various environmental samples, such as water (Leser et al., 1995) and rumen digesta (Koike & Kobayashi, 2001).

Ruminant livestock plays a significant role in global food security and nutrition. However, Ruminant production systems face serious challenges, in particular, because of their impact on GHG emissions. Animal product demand will elevate with the rapidly increasing global population. Thus, the environmental impact per unit of animal products will extend. Therefore, sustainable and immediate mitigation strategies are in high demand (Haque, 2018). This study aims to investigate the effect of the substitution of wheat bran by the PSP on rumen microbiota and gas production parameters in goats and camels by an in vitro approach.

## 2 MATERIALS AND METHODS

PSP was received from a pomegranate juice factory in Ferdows (South Khorasan, Iran) and dried in shadow. The same basic ratio was prepared for camels and goats by SRNS (2012). All ration components were ground in a heavy‐duty high‐rotation hammer mill to pass through a 1 mm mesh sieve to obtain the fine powder.

The experimental treatments were included: basic ration as control, basic ration + 5% PSP and basic ration + 10% PSP; PSP replaced wheat bran. The dry matter (DM), ash, CP, crude fat, neutral detergent fibre (NDF) and acid detergent fibre (ADF) were determined according to the procedure of the Association of Official Analytical Chemists (AOAC 2005) (Table 1).

**TABLE 1:** Ingredients and proximate composition of the diet.

	Diet		
Item Ingredients % of DM	1	2	3
Dehydrated alfalfa hay	20.2	20.2	20.2
Barley	35.1	35.1	35.1
Wheat bran	10	5	0
Pomegranate seed pulp	0	5	10
Straw	13.2	13.2	13.2
Beet pulp	18	18	18
Vitamin Minerals mix	1.8	1.8	1.8
Calcium carbonate	0.9	0.9	0.9
Salt	0.9	0.9	0.9
Metabolic energy (mcal/kg DM)	1.1	1.1	1.1
Crude protein % DM	12.9	12.8	12.8
ADF % DM	22.8	23.8	24.8
NDF % DM	47.7	48.3	49
Crude fat % DM	1.1	1.5	2
Ash % DM	10	9.9	9.8
Calcium % DM	1.1	1.1	1
Phosphor % DM	0.8	0.6	0.5

Abbreviations: ADF, acid detergent fibre; DM, dry matter; NDF, neutral detergent fibre.

In order to examine the effect of PSP on rumen microbial population and fermentation, the batch culture method was used. For three replications of in vitro gas production experiment, rumen fluids were collected from three slaughtered goats and two camels (fed in a pasture‐rearing system). These samples were immediately transported to the microbiology lab at the University of Birjand under anaerobic and thermal conditions (CO_2_ injection and 37°C) and filtered through four layers of cheesecloth into a glass amber container.

In vitro gas production was carried out using Blümmel et al. (1997) described. Buffer was added to rumen fluid at a 2:1 ratio and placed in the water bath at 39°C under continuous flushing with CO_2_. Then 50 mL of the solution was dispensed into each syringe containing 500 ± 10 mg DM feedstuffs and placed in the water bath at 39°C. The syringes were gently shaken every 2 h.

In each camel and goat species run, there were 45 samples of 3 treatments (with 15 replicates each) and 3 blank samples containing only buffered rumen fluid. Each of the 45 samples was randomly incubated 3 times.

The gas pressure and volume were recorded manually after 2, 4, 6, 8, 12, 24, 36, 48, 72, 96 and 120 h of incubation with a pressure transducer and plastic syringes. Volumes of gas production were recorded randomly (three replications for each treatment) until 120 h, and the cumulative gas values were fitted with the following exponential model without a lag phase, y=v×(l−exp−kt), where *y* is the cumulative gas volume (mL) produced at time *t* (h); *v* is the asymptotic gas volume (mL) and *k* is a constant rate (mL/h) (Schofield et al., 1994).

CH_4_, NH_3_‐N and pH, after 0, 12, 24, 48 and 72 h of incubation, were sampled from the culture medium with three replications for each parameter. Methane concentrations were determined by injecting 2 mL of gas into a gas chromatograph (Radpaya). Moreover, DM digestibility was sampled after 0, 12, 24, 48, 72 and 120 h of incubation with three replications for each treatment.

The content of each syringe was filtered through filter bags, and the residue was used to determine the degradability of the feed substrate. The pH value of the culture was measured using a pH meter. The content of NH_3_‐N was determined using phenol–hypochlorite (Broderick & Kang, 1980). The filtered sample at 12, 24 and 48 h of incubation was aliquoted into different tubes and kept at −20°C until DNA extraction.

The concentrations of VFAs in 24 h of incubation were measured according to Getachew et al. (1998) method; moreover, the efficiency of microbial protein synthesis in the rumen was estimated by the method described by Blümmel et al. (1997).

For molecular microbial analyses, the DNA of the culture fluid was extracted according to the modified RBB + C method (Yu & Morrison, 2004). The concentration of DNA was determined in absorbance at 260 and 280 nm using a NanoDrop Thermo 2000 spectrophotometer. The DNA samples were stored at −20°C until analysis.

Multiple alignments of the 16s rRNA and 18s rRNA gene sequences were used to identify conserved regions along with rumen methanogen and protozoa sequences, respectively. Several primer sets, including degenerate and non‐degenerate, were designed using Oligo (MBInsights) and Genetyx software (Software Development) from these conserved regions to amplify target and competitive fragments (Table 2).

**TABLE 2:** The specific primers for polymerase chain reaction (PCR) and competitive PCR (cPCR).

Microorganism	Primer	Primer sequence	Length (kbp)
Protozoa	Target	F 5′‐TCAGTACCTTATGAGAAATC‐3′	360
		R 5′‐CAGGACATATAAGGGCATCAC‐3′	
	Competitor	F 5′‐TCAGTACCTTATGAGAAATC‐3′	235
		RC 5′‐CAGGACATATAAGGGCATCACGACAAATCACTCCACCAACTA‐3′
methanogen	Target	F 5′‐AGTCAGGCAACGAGCGAGAC‐3′	296
		R 5′‐GTGTGTGCAAGGAGCAGGGAC‐3′	
	Competitor	FC 5′‐AGTCAGGCAACGAGCGAGACGCWACACGCGGGCTACAATG‐3′	196
		R 5′‐GTGTGTGCAAGGAGCAGGGAC‐3′	

*Note*: Length: the amplified fragment size by primers, F: forward primer, R: reverse primer, FC: forward primer for competitor, RC: reverse primer for competitor.

Competitor DNA for methanogen (196 kb) and protozoa (235 kb) was produced by removing a fragment to yield a shorter fragment.

Cycle conditions for cPCR were: (a) for protozoa population: 95°C for 9 min, 30 cycles; 95°C for 40 s, 55°C for 40 s, 72°C for 40 s and then 72°C for 10 min; (b) for methanogen population: 95°C for 9 min, 34 cycles; 95°C for 40 s, 62°C for 40 s, 72°C for 40 s and final extension at 72°C for 10 min.

**FIGURE 1:**
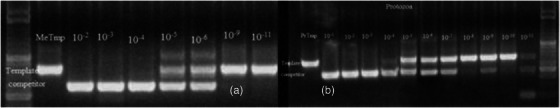
Competitive polymerase chain reaction (cPCR) product of competitor DNA dilutions with target DNA: methanogen population (a), protozoa population (a). MeTmp: Template DNA for methanogen, PrTmp: Template DNA for protozoa, 10^−1^ to 10^−11^: suitable dilutions for counting population.

To determine the sensitivities of cPCR assay, competitor fragments from the PCR product for both populations were serially diluted. In the following step, cPCR reactions were carried out in a final volume of 25 μL, consisting of 1 μL of each dilution, 1 μL of target DNA, 12.5 μL Master Mix 2x (Yekta Tajhiz Azma), 10 pM as a forward primer, 10 pM as a reverse primer and 8.5 μL distilled water. In order to distinguish suitable dilutions for the counting population, the PCR products were analysed by running on 1% agarose gels (Figure 1a for methanogen population and B for protozoa population).

Each competitor was co‐amplified by PCR with total DNA from each culture. Negative controls (DNA was replaced by water) were run for each set. The cPCR products were separated on a 1% agarose gel containing ethidium bromide and photographed. The negatives were scanned, and band intensities were measured using image analysis software (ImageJ). Then, using the following formula, the number of copies of the competitor fragment in the specified dilutions was obtained (Ball et al., 2013). In the next step, the intersection points were plotted, and by performing mathematical calculations, the frequency of microbial populations at different times and in different animals was calculated.

$$\begin{array}{ccc} & & \mathrm{Number}\;\mathrm{of}\;\mathrm{copies}\;=\;\mathrm{amount}\times \;6.022\;\times 10^{23})/\\ & & \;\left(\mathrm{length}\;\times \;1\times 10^{9}\times \;650\right) \end{array}$$

$$\mathrm{Number}\;=\;\left(\mathrm{ng}\times \frac{\mathrm{number}}{\mathrm{mole}}\right)/\left(\mathrm{bp}\times \frac{\mathrm{ng}}{g}\times \frac{g}{\mathrm{mole}\;\mathrm{of}\;\mathrm{bp}}\right)$$

### 2.1 Statistical analysis

The data were analysed in the mixed procedure (SPSS, 2009) to test the differences among three treatments, two species and three runs in ruminal fermentation characters and the population of the total methanogens and protozoa. Treatments, time, species and their interactions were included as fixed factors, and incubation was run as a random factor using the following model:

$y_{ijk}=\mu +T_{i}+R_{j}+S_{k}+b\left(Y_{ijk}-{\underset{-}{Y}}_{0}\right)+e_{ijk}$, where $y_{ijk}$ is the dependent variable, *μ* is an overall mean, *T_i_* is the treatment, *R_j_* is run, *S_k_* is the species and *e_ijk_* is a random error. The average of 3 replicate bottles per treatment per run per animal was considered the experimental unit. The Tukey test was used to test differences among individual means for significance. Significance was declared at *p* ≤ 0.05.

## 3 RESULTS

### 3.1 Effects on rumen gas production

According to the results of Table 3, in 120 h, the addition of PSP did not affect total gas and constant rate (*p* > 0.05); however, the asymptotic gas volume, *G*_0.5_ (mL) and CH_4_ percentage decreased by increasing the concentration of pomegranate seed oil (PSO). Adding 10% PSP showed the lowest constant gas rate and methane production (*p* < 0.05). All kinetic parameters, except for *K*, in different species, were affected by adding PSP, and their amount was higher in goats than in camels (*p* < 0.05). The camels produced the lowest and the goats the highest percentage of CH_4_ (*p* < 0.05).

**TABLE 3:** Effect of pomegranate seed pulp (PSP) in different treatments and animals on gas production kinetics and methane production.

Item		*V* (mL)	*K* (%)	*T*_0.5_ (h)	*G*_0.5_ (mL)	Gas (mL)	Methane/total gas (%)
Treatment^*^	1	128.1^a^	0.1	11.1	64^a^	94.5	11.4^a^
	2	124.2^ab^	0.1	10.4	62.1^ab^	94	10.1^ab^
	3	118.1^b^	0.1	10.1	59.1^b^	90.5	8.1^a^
Significant		0.02	0.2	0.1	0.02	0.2	0.02
SEM		2.54	0.002	0.36	1.26	1.66	1
Animal	Goat	141.2^a^	0.1	9.9^b^	70.6^a^	106.6^a^	7.3^b^
	Camel	105.8^b^	0.1	11.2^a^	52.9^b^	79.5^b^	12.5^b^
Significant		0.0001	0.9	0.001	0.0001	0.0001	0.0001
SEM		2.07	0.001	0.3	1.03	1.35	0.9

*Note*: This means that a column without a common superscript letter differs (*p*   <  0.05). Parameters of gas production kinetics were estimated using the model proposed by Schofield et al. (1994). Gas: total gas production, *G*_0.5_: fractional rate of gas production at half‐time, *T*_0.5_: half‐time of gas production, *V*: asymptotic gas volume, *K*: constant rate.

* Treatments were included: basic ration as control (1), basic ration + 5% PSP (2) and basic ration + 10% PSP (3); PSP replaced wheat bran.

### 3.2 Effects of treatments on ruminal fermentation characters

As shown in Table 4, in 12, 24 and 48 h, the concentration of NH_3_‐N and microbial protein demonstrated a linear increase by the addition of PSP in the cultures; the highest NH_3_‐N and MP were observed for the highest percentage of PSP (*p* < 0.05) but did not affect pH value, total VFA and, the proportion of acetate, butyrate and propionate in the cultures (*p* > 0.05).

**TABLE 4:** Effect of pomegranate seed pulp (PSP) on fermentative characters in ruminal culture.

	Treatment^*^				Animal		
Measurement	1	2	3	*p* Value	Goat	Camel	*p* Value
pH	6.3 ± 0.08	6.4 ± 0.08	6.4 ± 0.08	0.419	6.2^b^ ± 0.08	6.6^a^ ± 0.08	0.0001
NH_3_‐N (mg/dL)	29.8^b^ ± 0.78	30.5^b^ ± 0.77	32.4^a^ ± 0.77	0.029	39.1^a^ ± 0.73	22.7^b^ ± 0.85	0.0001
MP (mg/mL)	121^b^ ± 9.55	126^b^ ± 9.44	156.5^a^ ± 8.88	0.005	113.1^b^ ± 8.93	157.9^a^ ± 8.36	0.003
Total VFA (mM/mL)	2.2 ± 0.04	2.2 ± 0.04	2.1 ± 0.04	0.191	2.5^a^ ± 0.03	1.9^b^ ± 0.03	0.0001
Acetate (mM/mL)	0.5 ± 0.08	0.5 ± 0.08	0.4 ± 0.07	0.550	0.4 ± 0.07	0.5 ± 0.07	0.446
Propionate (mM/mL)	0.2 ± 0.04	0.2 ± 0.04	0.2 ± 0.03	0.875	0.2 ± 0.03	0.2 ± 0.03	0.713
Butyrate (mM/mL)	0.3 ± 0.05	0.3 ± 0.05	0.4 ± 0.04	0.313	0.3 ± 0.44	0.3 ± 0.41	0.326
DDM	0.3 ± 0.005	0.3 ± 0.005	0.3 ± 0.005	0.169	0.2^b^ ± 0.004	0.3^a^ ± 0.005	0.013
NDFD (%)	44^ab^ ± 1.66	48^a^ ± 1.64	40^b^ ± 1.64	0.002	44 ± 1.40	44 ± 1.67	0.985

*Note*: Means in a row without a common superscript letter differ (*p*  <  0.05). The data were presented as mean ± SEM.

Abbreviations: DDM, digestible dry matter; MP, microbial protein; NDFD, neutral detergent fibre digestibility; VFA, volatile fatty acid.

^*^Treatments were included: basic ration as control (1), basic ration + 5% PSP (2), and basic ration + 10% PSP (3); PSP replaced wheat bran.

Furthermore, different species affected the pH value and the concentrations of NH_3_‐N, MP and total VFA (*p* < 0.05). The camels had the highest (*p* < 0.05) pH and MP concentrations and the lowest NH_3_‐N and total VFA concentrations compared with goats. Animal species did not affect the proportion of acetate, butyrate and propionate in the ruminal cultures (*p* > 0.05).

DM digestibility in different treatments was not affected. However, the digestibility of NDF in diets containing different levels of PSP was affected by animal species in Table 5 (*p* < 0.05). The treatment containing 5% of PSP had the highest, and the treatment containing 10% of PSP had the lowest NDF digestibility (NDFD), which can be due to the increase in fat percentage, according to Table 1. NDFD was significantly affected by time (*p* < 0.05), and it was not affected by the two animals.

**TABLE 5:** Effect of pomegranate seed pulp (PSP) in different treatments and animal species on the population of ruminal methanogen and protozoa.

Factor			Protozoa (log_10_ copies per mL)	Methanogen (log_10_ copies per mL)
Treatment^*^	1		5.1	4.9
	2		5	5
	3		5	5
SEM			0.11	0.11
*p* Value			0.7	0.8
Species	Goat		5.4^a^	5.3^a^
	Camel		4.7^b^	4.6^b^
SEM			0.09	0.08
*p* Value			0.0001	0.0002
Treatment × species	Camel	1	4.8	4.5
		2	4.7	4.7
		3	4.7	4.6
	Goat	1	5.5	5.3
		2	5.4	5.3
		3	5.3	5.4
SEM			0.17	0.15
*p* value			0.9	0.6

*Note*: Means within a column with unlike superscripts differ (*p* < 0.05).

Abbreviation: SEM, standard error of the mean.

* Treatments were included: basic ration as control (1), basic ration + 5% PSP (2) and basic ration + 10% PSP (3); PSP replaced wheat bran.

Compared with the control (Table 4), treatment including 5% PSP significantly increased the NDFD, whereas treatment containing 10% PSP reduced the NDFD (*p* < 0.05). NDFD was not affected by different species (*p* > 0.05). In contrast, the digestibility of DM was highest for camels compared with goats (*p* < 0.5), and the addition of PSP did not change digestible dry matter (*p* > 0.05).

### 3.3 Effect on microbial populations

The results of agarose gel show that by decreasing the abundance of competitor fragments in the PCR reaction product, the pattern fragment has more opportunity for amplification by primers (which decreases the rival band intensity and increases the pattern fragment band intensity). After analysis by ImageJ software and methods, a linear diagram was described, and the intersection point was determined for each sample of camel and goat rumen fluid under the effect of treatments and at times 12, 24 and 48.

**FIGURE 2:**
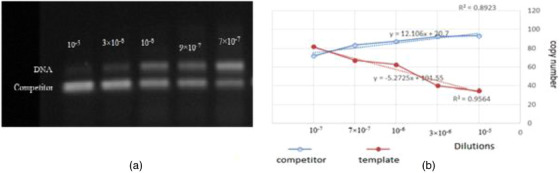
An example of (a) the optical image of the competitive polymerase chain reaction (cPCR) amplification and (b) its linear graph for qualification of the microbial population in culture.

Co‐amplification of the template with competitor DNA showed a linear relationship between the concentration of competitor and DNA. The intersection points were plotted, and the frequency of microbial population was calculated. Figure 2 shows an optical image of the cPCR reaction product (Figure 2a) and the linear graph (Figure 2b) to qualify the microbial population in a ruminal culture.

According to the results of Table 5, protozoa and methanogen populations were not affected by different treatments in 24 h. However, numerically, the treatment containing 5% of PSP had the lowest number of protozoa and methanogens compared to the control treatment, probably due to tannins. The animal significantly affects the change of microbial populations (*p* < 0.05). The population of protozoa and methanogens in goats is higher than in camels.

The most abundant protozoa and methanogen populations detected (Table 6) were in goats (*p* < 0.05). Different treatments and interactive effects between treatment and species did not affect the methanogen and protozoa populations (*p* > 0.05). The correlation between the two populations (Table 6) was 46% (*p* < 0.05).

**TABLE 6:** Correlation between the population of methanogen and protozoa.

Population	Methanogen (log_10_ copies per mL)	Protozoa (log_10_ copies per mL)
Methanogen (log_10_ copies per mL)	1	0.461
Significant	0.0004	
Protozoa (log_10_ copies per mL)	0.461	1
Significant	0.0004	

## 4 DISCUSSION

### 4.1 Effect of treatment on ruminal fermentation and microbiota

Recently, several studies have been conducted to distinguish the effect of pomegranate by‐products on rumen microbial fermentation (Abarghuei et al., 2013; Razzaghi et al., 2015; Taher‐Maddah et al., 2012). In the present study, an in vitro assay was used to investigate the effect of PSP on ruminal fermentation and microbial population. Our result indicated that asymptotic gas, fractional gas production rate at half‐time, CH_4_ production and NDFD were linearly decreased by increasing the PSP percentage. Natalello et al. (2020) and Mirzaei‐Aghsaghali et al. (2011) reported that treatment containing pomegranate seed inhibited gas production by reducing the microbial population and fibre digestibility. Kamalak et al. (2004) reported that there is a negative correlation between cell wall compositions (NDF, ADF, structural carbohydrates and tannins) and estimated parameters of gas production. According to Ko et al. (2021), pomegranate rind is a rich source of dietary fibre (17.33%–27.84% w/w) and pectin (6.8%–10.1% w/w). PSP contains some bioactive compounds (anthocyanins, flavonoids, tannins etc.), which have been determined to possess anti‐microbial and anti‐oxidant properties (Heber, 2011; Ko et al., 2021; Viuda‐Martos, 2010). It has been shown that pomegranate peel is rich in tannin, which has a negative effect on gas production and ruminal fermentation (Feizi et al., 2005). Moreover, tannins have been demonstrated to reduce ruminal microorganism activity and highly influence ruminal fermentation (Vasta et al., 2019). The mechanisms proposed so far to explain tannin antimicrobial activity include inhibition of extracellular microbial enzymes, deprivation of the substrates required for microbial growth, direct action on microbial metabolism through inhibition of oxidative phosphorylation, metal ions’ deprivation or the formation of complexes with the cell membrane of bacteria, causing morphological changes of the cell wall and increasing membrane permeability (Liu et al., 2013; Scalbert, 1991). Indeed, tannins are multidentate ligands that may form strong complexes with proteins and inhibit bacteria metabolism (Sharifi et al., 2019).

Ruminal CH_4_ emission is strongly associated with food intake and diet components. Thus, dietary intervention strategies such as supplementation of feed with agricultural by‐products can affect the amount of CH_4_ emitted (Histrov et al., 2013; Ugbogu et al., 2019). Long‐chain fatty acid and long‐chain unsaturated fatty acid impair CH_4_ production and feed degradation in ruminants by a physical coating of feed particles and limiting fibre digestion, inhibition of protozoa, a toxin to methanogens, and reduction of hydrogen during biohydrogenation in the rumen (Martin et al., 2010; Martin et al., 2008; Hook et al., 2010). On the other hand, the complex formed by tannin with protein and carbohydrates appears to be a reason for the decline in CH_4_ emission and feed digestibility (Mueller‐Harvey, 2006). McSweeney et al. (2001) demonstrated that any reduced access to nutrients reduces microbial fermentation, fibre degradation and, consequently, CH_4_ formation. Our results are comparable to Giller et al. (2021) and Maleki et al. (2016), who reported that pomegranate by‐products might be an option for methane‐mitigating feed supplements, which might be beneficial to nutrient utilization and growth in ruminants.

In this study, concentrations of ruminal NH_3_‐N and MP were increased by inclusion levels of PSP. An increase in ammonia‐nitrogen production will benefit the increase in the protozoa population and the decomposition of microbial protein (Busquet et al., 2006). Nevertheless, comparison with the literature is challenging because information on the effect of pomegranate peel, seed and oil revealed a decreased concentration in rumen fluid of ammonia (Natalello et al., 2020; Refat et al., 2015; Sharifi et al., 2019). The impact of tannins on ruminal protein metabolism has been attributed to their ability to attach plant protein, decline the microbial enzyme's activity, reduce bacteria growth rate and finally decrease ruminal NH_3_‐N (Molan et al., 2001; Min et al., 2005). Pomegranate rind is rich in polyphenolic components, the majority of which are tannins consisting of punicalin, ellagic acid, gallagic acid and punicalagin (Gil et al., 2000). The inclusion of antioxidants in the diet ameliorates the adverse effects by neutralizing peroxides and reducing the peroxidation of fatty acids (Vázquez‐Añón & Jenkins, 2007); furthermore, this way leads to enhanced rumen health and microbial efficiency (Abarghuei et al., 2014).

However, dry matter digestibility (DMD) was not affected when measured as substrate disappearance from cultures. Additionally, other parameters related to the extent of ruminal feed degradation, such as total gas, the concentrations of total and individual VFA, pH and microbial population, were not affected by the addition of PSP in diets. Similarly, a report has proven that the concentration of total VFA and the molar proportion of individual VFAs were not affected by the inclusion of pomegranate peel extract in the diet (Abarghuei et al., 2013). Safari et al. (2018) also reported that DM intake was not affected by supplementation with either PS or PSP.

Due to the additon a pomegranate by‐product, Jami et al. (2012) observed an increase in abundance in lactic acid bacteria and methanogen archaea communities; moreover, a decrease in abundance in main cellulolytic bacterial species. Abarghuei et al. (2014) and Rajabi et al. (2017) suggested that PPE supplementation has reduced total protozoa enumeration. Maleki et al. (2016) indicated that total bacteria and protozoa counts increased with rising PSO levels, whereas the methanogen population declined significantly.

No conclusive explanation could be found from comparing studies about the effect of pomegranate on the microorganism population in a rumen due to variations in the diet type, sampling method, different types and levels of pomegranate supplements, species and other animal conditions.

### 4.2 Effect of species on ruminal fermentation and microbiota

Although ruminant animals can digest fibre via microbial fermentation to obtain helpful energy needed for various biological functions, however, our results revealed that this ability could be influenced by ruminant species. Henderson et al. (2015) showed that the composition of the rumen microbiota is primarily determined by diet, and it is likely less influenced by the host ruminant. We found that the amount of DM digestibility, NDFD, microbial protein and pH in the camels were higher than in the goats.

Iqbal and Khan (2001) declared that camels have a unique physiological system that permits them to feed on thorny plants. Furthermore, camelids have a lower feed intake compared to other ruminants. True ruminants’ digestive tract anatomy differs from camels, with a forestomach differentiated into three compartments (Von Engelhardt et al., 2007). Gharechahi et al. (2015) reported that the camel rumen microbiome was structurally similar but compositionally distinct from other ruminants. The uniqueness of the camel rumen microbiome was related to enrichment for cellulolytic bacteria; therefore, they can digest highly fibrous plants. The retention time of substrate in camel rumen is longer than in other ruminants; thus, it improves the enzymatic efficiency of microbes, which is a prerequisite for effective fibre digestion (Samsudin et al., 2012). McSweeney et al. (1989) demonstrated that buffalo spend 50% more time on rumination than cattle, which results in a 30% lower residence time for substrate matter in the rumen. Sponheimer et al. (2003) reported that South American camelids have higher efficiency in DM and fibre digestion than goats, which is possibly due to their relatively longer particulate matter mean retention times.

Our results revealed a higher protozoa and methanogen population in the goats than in the camels. Previous studies have reported differences in microbial population structure between different species. King et al. (2011) found a potential difference in the population structure of methanogen communities between Holsteins and Jerseys. Crowley et al. (2017) suggested that no microbial community is common to caecotrophagic animals. Denman and McSweeney (2006) demonstrated significant differences in the microbial population of the rumen of different species.

Due to the lower fibre content of the diet and metabolism, Dittmann et al. (2014) demonstrated that camelids produce clearly less CH_4_ than ruminants. Danielsson et al. (2017) indicated that the correlation between archaea with CH_4_ emissions has been weak. Bhatta et al. (2013) reported that ciliates are involved in methanogenesis via their abundant H_2_ production. Tan et al. (2011) reported that the elimination of protozoa from the rumen decreased methane production, which seems to be due to the symbiosis relationship between protozoa and methanogen. A report has proved that smaller ciliate and their associated methanogens will be more active in methanogenesis than larger species (Ranilla et al., 2007). Host animal has a controlling effect on their rumen microbiota (King et al., 2011). Additionally, metagenomics has revealed that the abundance of specific groups of microbial genes can be highly predictive of CH_4_ emissions (Wallace et al., 2015).

In this study, total gas production and NH_3_‐N were significantly higher in the goats than in the camels, which is probably related to the population of protozoa and methanogens (Table 5). Chanthakhoun et al. (2012) reported that the difference in gas production in buffalo and cattle was attributed to the rumen microorganism's population difference. Protozoa elimination in the rumen decreased rumen ammonia concentration and seems to be due to decreased bacterial protein breakdown and feed protein degradability in the lack of rumen protozoa (Newbold et al., 2015), furthermore making the rumen more efficient in protein synthesis (Koenig et al., 2000).

We found that total VFA concentration was lower for the rumen fluid of camels than for goats. Lignocellulose degradation and fermentation by rumen microbes to form VFAs are very important for the nutrition of the ruminant (Aluwong et al., 2010). Species belonging to the phyla *Bacteroidetes, Firmicutes* and *Fibrobacteres* significantly contribute to the decomposition of lignocellulosic materials (Krause et al., 2003). Bhatt et al. (2013) indicated *Bacteroidetes* (55.5%) and *Firmicutes* (22.7%) phyla as predominant camel rumen taxa. Do et al. (2018) reported that the most abundant phylum in the rumen goats was *Bacteroidetes* (86.2%), and the second phylum was *Firmicutes* (9.5%). The molar proportion of individual VFAs was not affected by species. VFAs can build up in the rumen and reduce ruminal pH (Dijkstra et al., 2012). Lower pH in the goat's rumen was related to higher total VFA production than for the camels.

## 5 CONCLUSION

The present study concluded that PSP reduced methane emission and increased microbial protein, and it could be a good food industrial by‐product for ruminant nutrition, but more research is required. On the other hand, the rumen of goats had a higher methanogen and protozoa population than those in camels. Even though similar diets add to the cultures and are kept with the same condition. Moreover, the competitive PCR method effectively calculated the population of methanogen and protozoa.

## AUTHOR CONTRIBUTIONS

All authors contributed to the design and implementation of the research, the analysis of the results and the writing of the manuscript.

## CONFLICT OF INTEREST STATEMENT

The authors declare that they have no known competing financial interests or personal relationships that could have appeared to influence the work reported in this paper.

## FUNDING INFORMATION

University of Birjand

## ETHICS STATEMENT

The rumen fluid collection was conducted on animals after their slaughtering to avoid interfering with the welfare of animals.

### PEER REVIEW

The peer review history for this article is available at https://www.webofscience.com/api/gateway/wos/peer‐review/10.1002/vms3.1297.

## Data Availability

The datasets generated during the current study are available from the corresponding author upon reasonable request.
